# The prognostic influence of histological subtypes of micropapillary tumors on patients with lung adenocarcinoma ≤ 2 cm

**DOI:** 10.3389/fonc.2022.954317

**Published:** 2022-08-10

**Authors:** Liangdong Xu, Hangcheng Zhou, Gaoxiang Wang, Zhining Huang, Ran Xiong, Xiaohui Sun, Mingsheng Wu, Tian Li, Mingran Xie

**Affiliations:** ^1^ Department of Thoracic Surgery, Affiliated Provincial Hospital of Anhui Medical University, Hefei, China; ^2^ Department of Thoracic Surgery, The First Affiliated Hospital of University of Science and Technology of China (USTC), Division of Life Sciences and Medicine, University of Science and Technology of China, Hefei, China; ^3^ Department of Pathology, The First Affiliated Hospital of University of Science and Technology of China (USTC), Division of Life Sciences and Medicine, University of Science and Technology of China, Hefei, China

**Keywords:** non-small cell lung cancer, micropapillary component, sublobar resection, prognosis, survival

## Abstract

**Objective:**

This study aimed to explore the value of micropapillary histological subtypes in predicting the specific surgical specificity and lymph node metastasis prognosis of early lung adenocarcinoma.

**Methods:**

A total of 390 patients with lung adenocarcinoma were included who underwent surgery in the Department of Thoracic Surgery of the Affiliated Provincial Hospital of Anhui Medical University from January 2016 to December 2017. The data were analysed with SPSS 26.0 statistical software, and the clinicopathological data of the two groups were compared with the chi-square test. The survival rate was calculated by the Kaplan-Meier method, and the difference in survival rate between groups was analysed by the log-rank test. Multivariate survival analysis was performed using the Cox model.

**Results:**

Univariate analysis of the clinicopathological data of the patients showed that the micropapillary histological subtype was significantly associated with the survival rate of patients (p=0.007). The clinicopathological data of the patients were substituted into the Cox model for multivariate analysis, and the results showed that the micropapillary histological subtype was an independent prognostic factor affecting the survival rate of the patients (p=0.009).The average survival time of Group A (micronipple composition > 5%) was 66.7 months; the 1-year, 3-year, and 5-year survival rates were 98.8%, 93.0%, and 80.9%, respectively.The survival of the lobectomy group was better than that of the sublobectomy group and the survival of patients with systematic dissection was better than that of patients with limited lymph node dissection. The average survival time of Group B (micronipple composition ≤ 5%) was 70.5 months; the 1-year, 3-year, and 5-year survival rates were 99.3%, 95.4%, and 90.6%, respectively. There was no difference in the survival rate between the lobectomy group and sublobectomy group, and there was also no difference in survival between systematic lymph node dissection and limited lymph node dissection, The survival rate of Group B was significantly better than that of Group A.

**Conclusion:**

The micropapillary histological component is an independent risk factor after surgery in patients with ≤2 cm lung adenocarcinoma. When the proportion of micropapillary components is different, the prognosis of patients is different when different surgical methods and lymph node dissections are performed. Lobectomy and systematic lymph node dissection are recommended for patients with a micropapillary histological composition >5%; sublobar resection and limited lymph node dissection are recommended for patients with a micropapillary histological composition ≤5%.

## Introduction

At present, lung cancer is the malignant tumor with the highest mortality rate in the world, of which non-small-cell lung cancer (NSCLC) accounts for 80%-85%, with the most common histological type being adenocarcinoma (1, 2). With the development of imaging technology and the widespread use of low-dose spiral CT, an increasing number of small pulmonary nodules (≤ 2 cm) are found and confirmed as early lung adenocarcinoma by postoperative pathology (3, 4). For resectable non-small-cell lung cancer, lobectomy and mediastinal lymph node dissection are still the main comprehensive treatments (5, 6). According to the new classification proposed by the International Association for Lung Cancer Research (IASLC), the American Thoracic Society (ATS) and the European Respiratory Society (ERS), lung adenocarcinoma can be divided into five histological subtypes. Studies have shown that lung adenocarcinoma dominated by acinar type shows a good prognosis, while micronipple-based lung adenocarcinoma is associate with poor prognosis (7–9).Whether patients with micropapillary histological subtypes of lung adenocarcinoma can benefit from sublobectomy has not been studied, and the relationship between micropapillary components and lymph node metastasis is unclear. In this study, we aimed to explore the value of micropapillary histological subtypes in predicting the specific surgical specificity and lymph node metastasis prognosis of early lung adenocarcinoma and to select the best surgical scheme for optimal individualized treatment and prognosis stratification.

## Methods

### Patient selection

This study retrospectively analysed 1403 patients with NSCLC who underwent surgery in the Department of Thoracic Surgery of the Affiliated Provincial Hospital of Anhui Medical University from January 2016 to December 2017. Inclusion criteria were: 1) patients with primary lung adenocarcinoma confirmed by postoperative pathology; 2) tumor size ≤ 2 cm; 3) postoperative pathological stage was pT1-2N0M0; and 4) R0 resection. Exclusion criteria were: 1) received neoadjuvant therapy; 2) patients with multiple nodules; 3) incomplete medical records. Based on the above criteria, a total of 390 patients with lung adenocarcinoma were included in this study.

This study was approved by the Ethics Committee of the Provincial Hospital affiliated with Anhui Medical University.

### Research content

The study involves a comparative analysis of general clinicopathological data of patients and the relationship between micropapillary histological subtype components and survival rate.

We compared the effects of different surgical and lymph node dissection methods on the survival rate of patients with different micropapillary histological subtypes.

### Surgery and lymph node dissection

The surgical methods include lobectomy and sublobectomy, and sublobectomy also includes wedge resection and segmentectomy.

The indications of sublobectomy are determined by the general physical state of the patient and CT findings. Sublobulectomy was performed for peripheral lesions located outside the parenchyma of the lung or for CT shadows dominated by ground glass nodules.Sublobectomy was performed in patients with poor cardiopulmonary function, combined with basic cardiopulmonary diseases, or who were too old to tolerate lobectomy, regardless of tumor size or presence of solid components on CT.wedge resection or segmentectomy depends on the tumor location and surgical skill of the surgeon.

According to the recommendation from the National Comprehensive cancer Network,systematic lymph node dissection included 6 groups of lymph nodes, of which 3 groups were intrapulmonary and hilar, and 3 groups included mediastinal lymph nodes (10). Systemic hilar and mediastinal lymph node dissection routinely explores and dissects the 2R, 3A, 3P, 4R, 7-10 groups of lymph nodes and intrapulmonary lymph nodes on the right side and routinely explores and dissects the 4 L, 5-10 group lymph nodes and intrapulmonary lymph nodes on the left side. Limited lymph node dissection includes regional lymph node dissection, lymph node sampling dissection or no lymph node dissection. Limited lymph node dissection is will be performed according to the tumor size, intraoperative pathology and overall physical status of the patient.

### Histological evaluation

The pathological staging was based on the IASLC TNM staging system (8^th^ edition) (11). The pathological sections of all patients were blind reviewed and reclassified by two senior clinical pathologists in our hospital. When there were differences in the diagnosis between the two physicians, they were re-examined and classified by another clinical pathologist.

According to the IASLC/ATS/ERS classification system of lung adenocarcinoma, lung adenocarcinoma can be divided into five subtypes: lepidic, acinar, papillary, micropapillary and solid. The percentage of each tissue subtype was recorded in increments of 5%. If micropapillary components accounted for 5% of the tumor, one subtype was considered to exist. The pattern with the largest proportion was defined as the histologically dominant pattern. In this study, micronipple composition > 5% was defined as Group A, and micronipple composition ≤ 5% was defined as Group B.

### Postoperative follow-up

Follow-up was carried out in two ways: outpatient regular follow-up and telephone follow-up. The patients were followed up with every 4 months for years 1-2, every half-year for years 3-5, and once a year from the 6^th^ year. The relevant clinical information (including chest and brain CT, bone scan, abdominal and adrenal ultrasound, etc.) and the survival of the patients were obtained.

Overall survival was defined as the time from surgery to death from any cause. The end point of follow-up was March 2022.

### Statistical analysis

The data were analysed with SPSS 26.0 statistical software, and the clinicopathological data of the two groups were compared with the chi-square test. The survival rate was calculated by the Kaplan–Meier method, and the difference in survival rate between groups was analysed by the log-rank test. Multivariate survival analysis was performed using the Cox model. P < 0.05 indicates that the difference is statistically significant.

## Results

### Patient characteristics

The clinicopathological data of 390 patients with ≤2 cm lung adenocarcinoma who underwent lung surgery were included in this study, including 86 patients in Group A and 304 patients in Group B. There were 14 patients with the predominant subtype of micropapillary in Group A, while those in Group B were not predominantly micropapillary. The proportion of the micropapillary dominant subtype in Group A was higher than that in Group B, and there was statistical significance in the main pathological subtypes (p < 0.001). There was no statistical significance in sex, age, smoking history, preoperative comorbidities, tumor location, maximum tumor diameter,visceral pleural invasion,surgical method, or lymph node dissection method (p>0.05). (**Table 1**).

**Table 1:** Baseline characteristics of the study population.

	Group A (n = 86)	Group B (n = 304)	*χ2*	P Value
Sex			0.068	0.794
Male	37	126		
Female	49	178		
Age, year			0.189	0.664
≤60	45	151		
>60	41	153		
Smoking history			2.094	0.148
Yes	16	38		
No	70	266		
Preoperative comorbidities			0.350	0.554
Yes	41	134		
No	45	170		
Tumor location			2.114	0.715
RUL	26	109		
RML	9	27		
RLL	10	45		
LUL	29	86		
LLL	12	37		
Tumor diameter, cm			3.333	0.068
≤1	21	106		
>1, ≤2	65	198		
VPI			2.867	0.090
Present	21	60		
Absent	65	244		
Operation type			2.546	0.111
Lobectomy	58	176		
Sublobectomy	28	128		
Lymph node dissection type			2.167	0.141
SLND	59	182		
LLND	27	122		
Histologically dominant pattern			87.833	<0.001
Lepidic	15	162		
Acinar	39	124		
Papillary	13	9		
Micropapillary	14	0		
Solid	5	9		

RUL, right upper lung; RML, right middle lung; RLL, right lower lung; LUL, left upper lung; LLL, left lower lung; VPI, visceral pleural invasion; SLND, systematic lymph node dissection; LLND, limited lymph node dissection; Preoperative complication includes high blood pressure, diabetes, arrhythmia, asthma, and so forth.

### Univariate and multivariate analysis of patient prognosis

Univariate analysis of the clinicopathological data of the patients showed that the micropapillary histological subtype was significantly associated with the survival rate of patients (p=0.007) (**Table 2**). The clinicopathological data of the patients were substituted into the Cox model for multivariate analysis, and the results showed that the micropapillary histological subtype was an independent prognostic factor affecting the survival rate of the patients (p=0.009) (**Table 2**).

**Table 2:** The prognostic factors associated with overall survival of patients in groups A, B by univariate analysis and multivariate Cox regression.

Variables	Case	Univariate		Multivariate	
		Mean survival time (month) (95% CI)	P Value	OR (95%CI)	P Value
Sex			0.946	–	0.949
Male	163	69.8			
Female	227	69.8			
Age, year			0.433	–	0.356
≤60	196	70.7			
>60	194	69			
Smoking history			0.361	–	0.461
Yes	54	69.2			
No	336	70			
Preoperative comorbidities			0.183	–	0.187
Yes	175	68.7			
No	215	70.7			
Tumor location			0.384	–	0.074
RUL	135	70.7			
RML	36	66.4			
RLL	55	65			
LUL	115	71.1			
LLL	49	70			
Tumor diameter, cm			0.273	–	0.182
≤1	127	68.8			
>1	263	70.3			
VPI			0.09	–	0.129
Present	81	68.4			
Absent	309	70.2			
Operation type			0.139	–	0.088
Lobectomy	234	70.5			
Sublobectomy	156	68.8			
Lymph node dissection type			0.94	–	0.875
SLND	241	70			
LLND	149	70.2			
Micropapillary component			0.007	0.436 (0.234-0.813)	0.009
>5%	86	66.7			
≤5%	304	70.5			

RUL, right upper lung; RML, right middle lung; RLL, right lower lung; LUL, left upper lung; LLL, left lower lung; VPI, visceral pleural invasion; SLND, systematic lymph node dissection;LLND, limited lymph node dissection; Preoperative complication includes high blood pressure, diabetes, arrhythmia, asthma, and so forth.

### Survival of patients in each group

**Figure 1:**
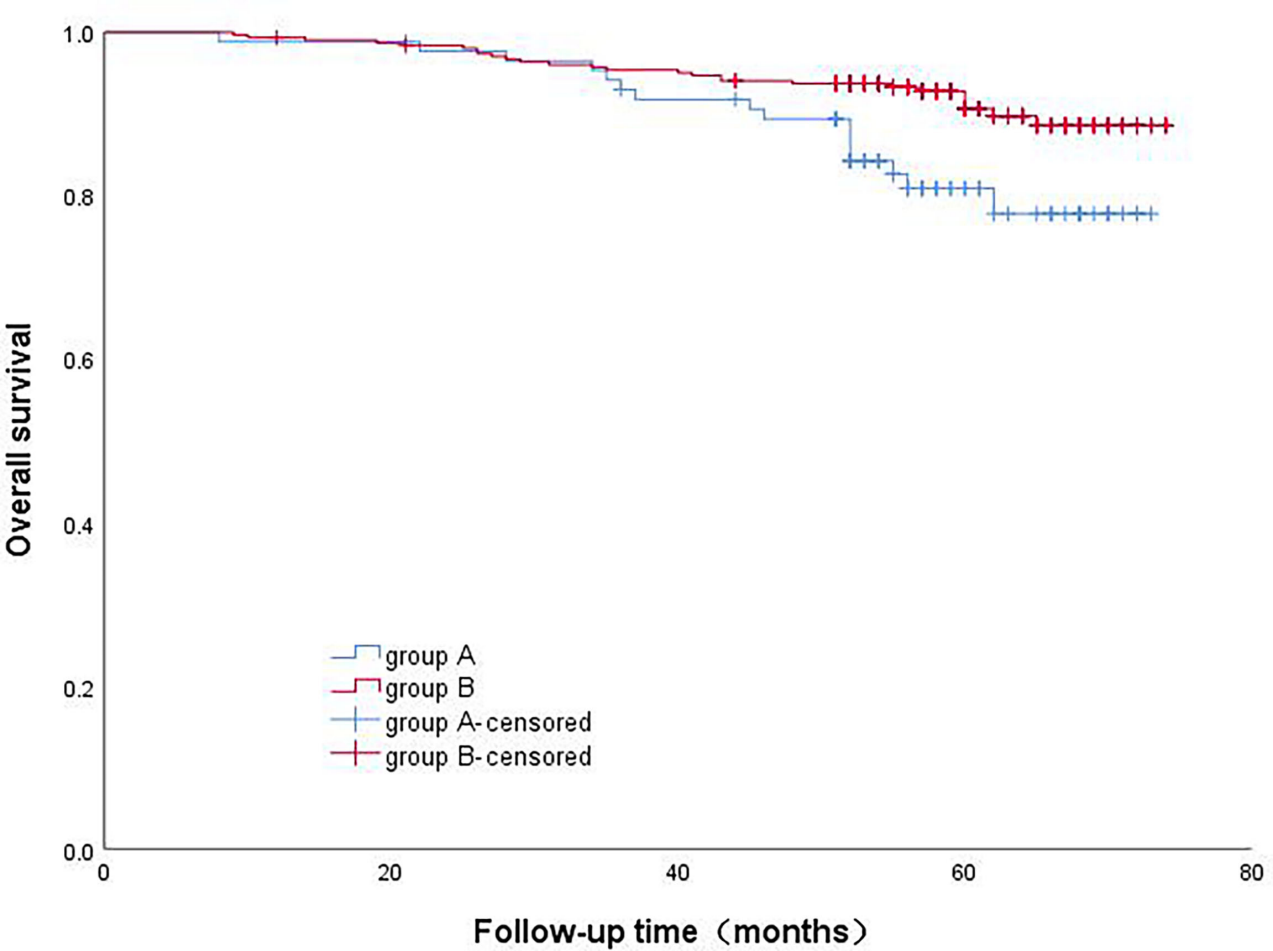
Comparison of patients survival rate with different micropapillary proportions.

A total of 390 patients were followed from January 2016 to March 2022, with a total follow-up period of 75.0 months and a median follow-up period of 57.0 months. Thirty-five patients were lost to follow-up. The average survival time of the whole group of patients was 69.8 months; the 1-year, 3-year, and 5-year survival rates were 99.2%, 94.8%, and 88.5%, respectively. The average survival time of Group A was 66.7 months; the 1-year, 3-year, and 5-year survival rates were 98.8%, 93.0%, and 80.9%, respectively, and the average survival time of Group B was 70.5 months; the 1-year, 3-year, and 5-year survival rates were 99.3%, 95.4%, and 90.6%, respectively. The survival rate of Group B was significantly better than that of Group A, and the result was statistically significant (p=0.007) (**Figure 1**).

### The prognostic effect of micropapillary histological components in patients with different surgical methods and different lymph node dissections

**Figure 2:**
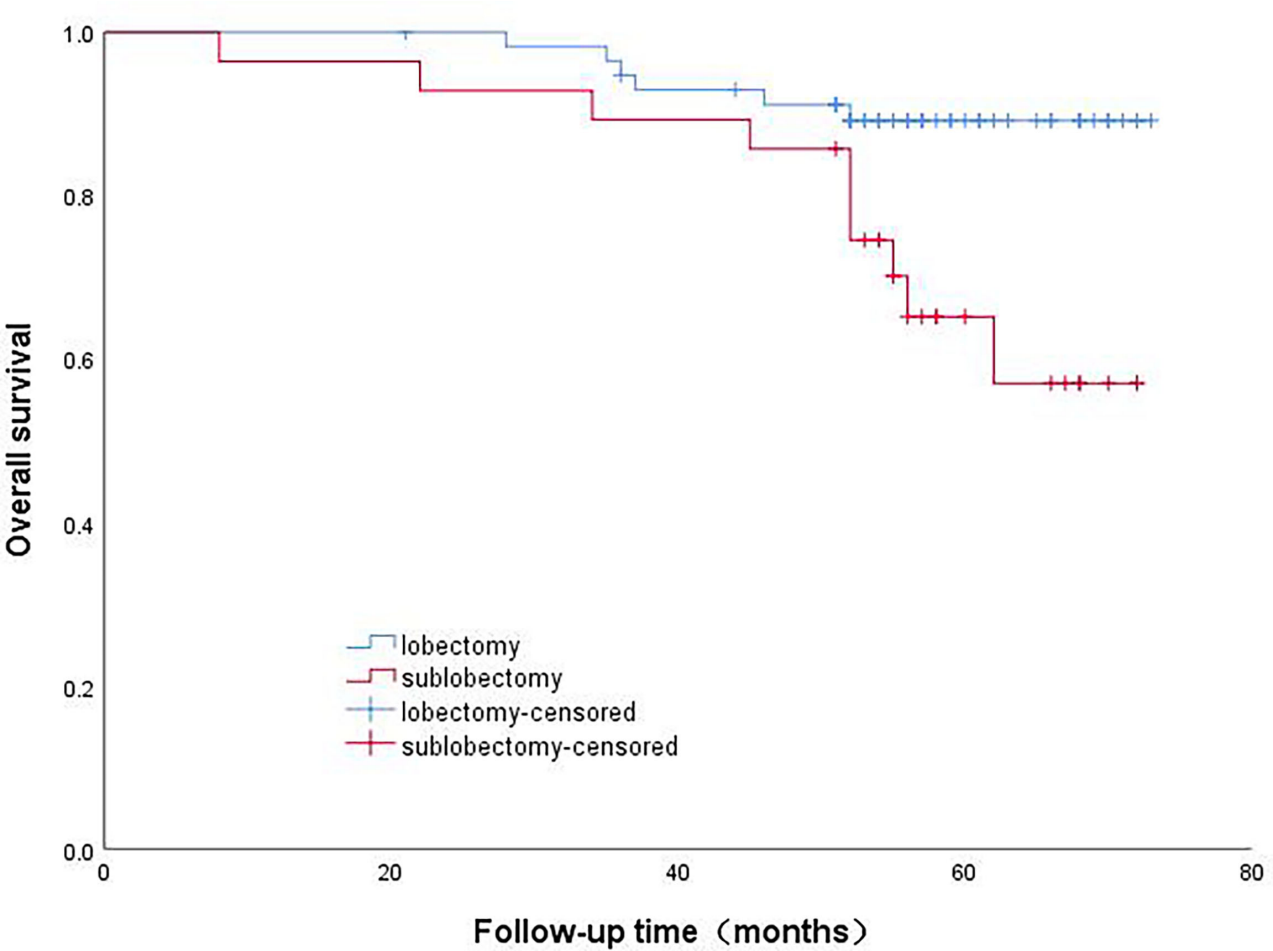
Survival curve of patients with micropapillary composition >5% with different surgical methods.

**Figure 3:**
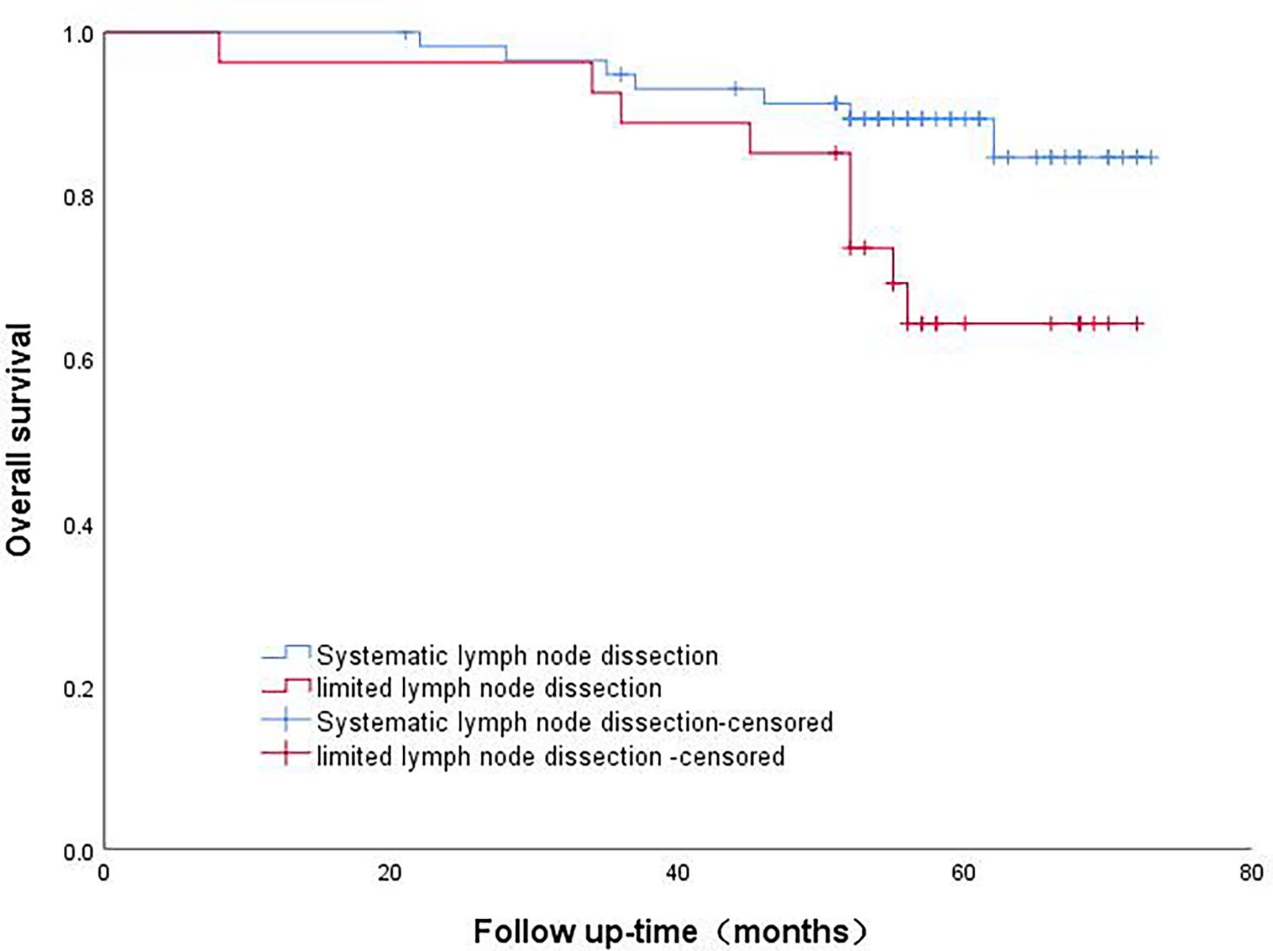
Survival curve analysis of patients with micropapillary component>5% with different lymph node dissection methods.

The patients in Group A were divided into the lobectomy group and sublobectomy group, and a survival curve analysis was performed. It was found that the survival of the lobectomy group was better than that of the sublobectomy group, and the result was statistically significant (p=0.008). (**Figure 2**) Systematic lymph node dissection and limited lymph node dissection were used for survival analysis. The survival of patients with systematic dissection was better than that of patients with limited lymph node dissection, and the result was statistically significant (p=0.028). (**Figure 3**).

**Figure 4:**
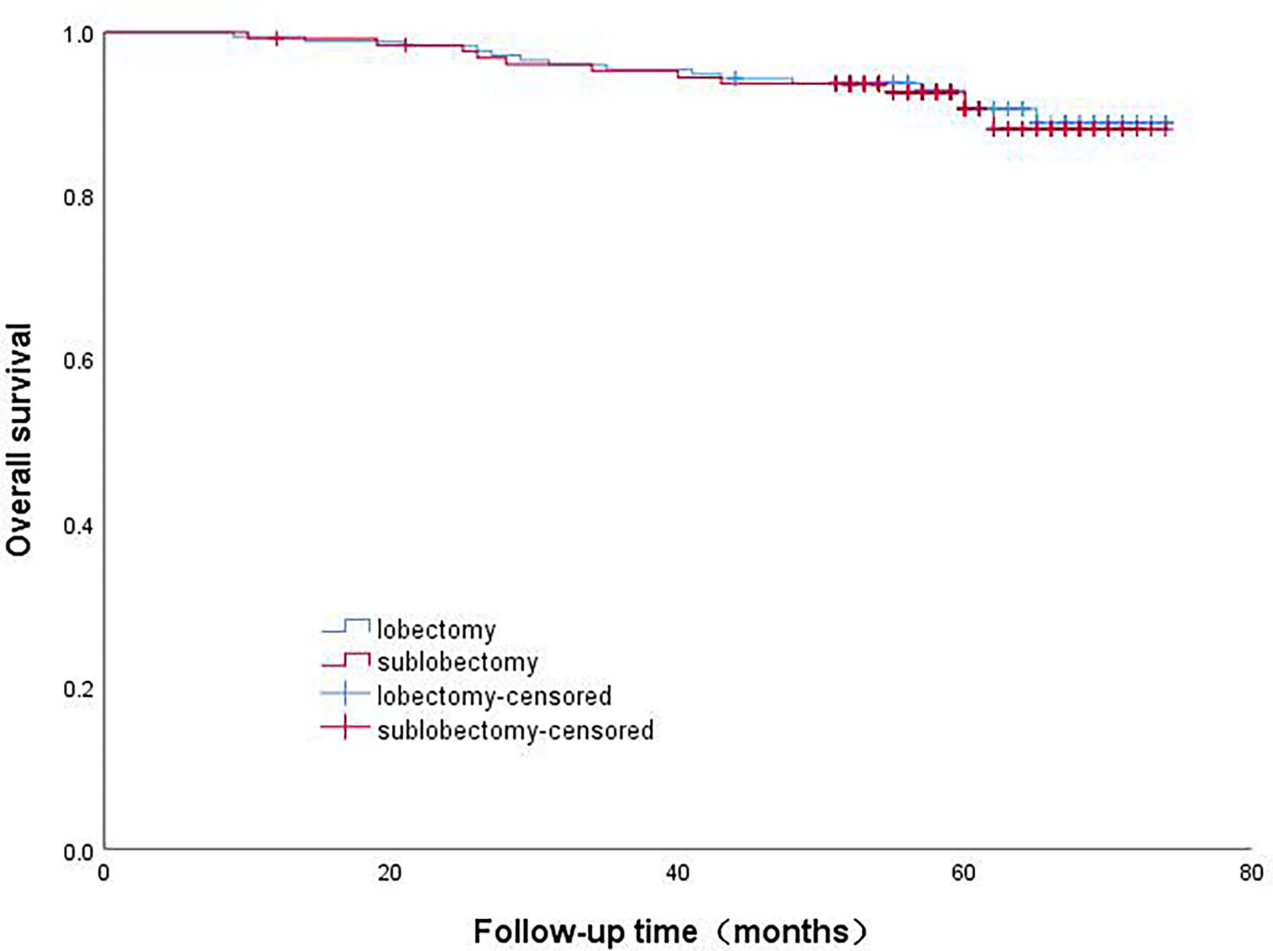
Survival curve of patients with micropapillary composition ≤ 5% with different surgical methods.

**Figure 5:**
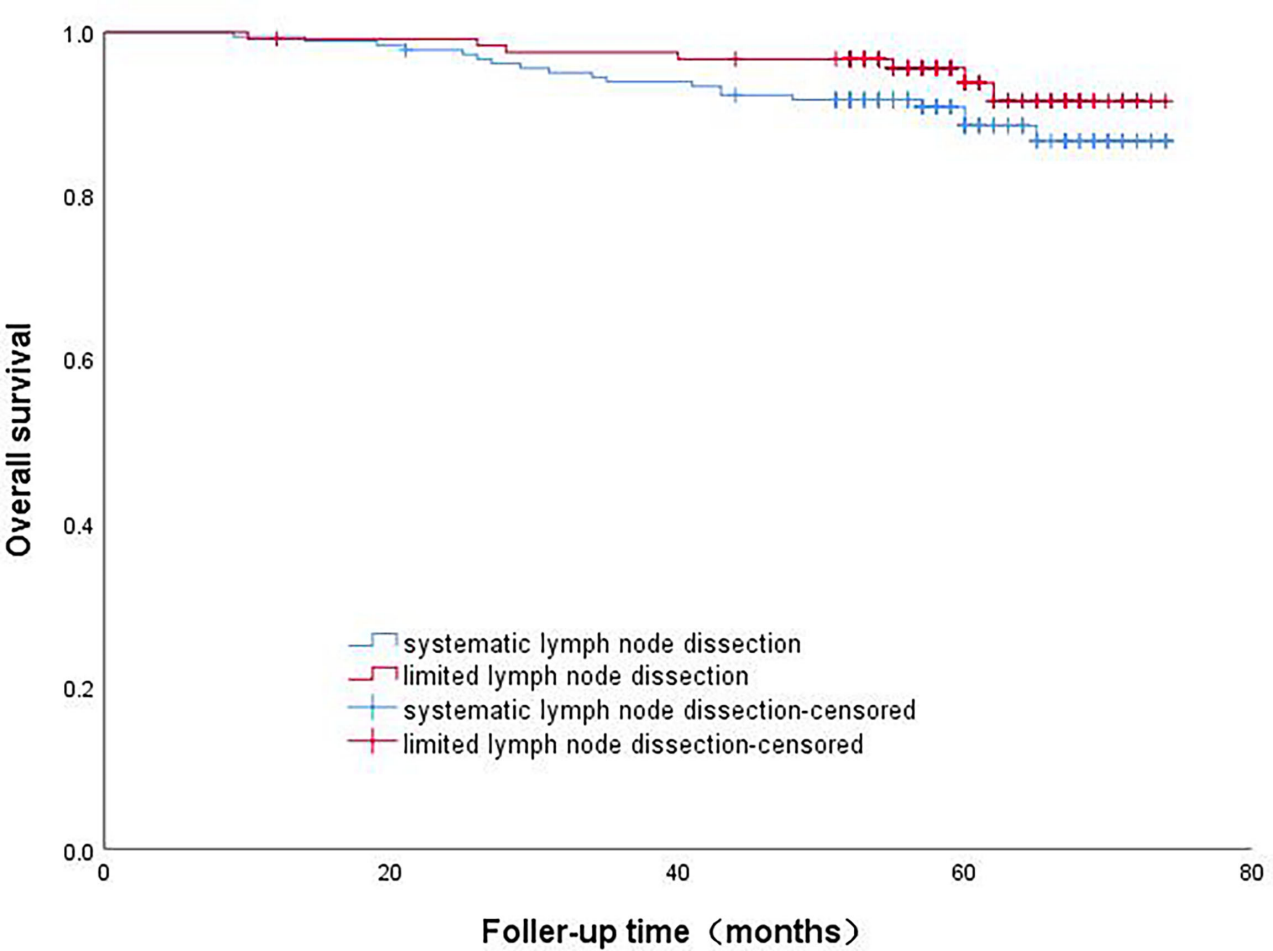
Survival curve analysis of patients with micropapillary component ≤5% with different lymph node dissection methods.

The patients in Group B were divided into the lobectomy group and sublobectomy group, and a survival curve analysis was carried out. There was no difference in the survival rate between the two groups, and the result was not statistically significant (p=0.844). (**Figure 4**) There was no difference in survival between systematic lymph node dissection and limited lymph node dissection, and the results were not statistically significant (p=0.159) (**Figure 5**).

## Discussion

The survival and prognosis of patients with lung cancer are related to many clinical and pathological factors. Many studies have reported that the micropapillary components of lung adenocarcinoma are closely related to lymph node metastasis, vascular tumor thrombus, visceral pleural invasion and airway diffusion (12, 13). This may be an independent risk factor for postoperative local metastasis and recurrence of early-stage NSCLC, and this is of high value for predicting the biological behaviour of tumours. This study also found that the micropapillary histological subtype component was an independent risk factor in patients with lung adenocarcinoma ≤ 2 cm after surgery, and when the proportion of micropapillary components was different, the prognoses of patients who underwent different surgical methods and lymph node dissection were different. Lobectomy and systematic lymph node dissection in patients with micropapillary histology >5% have better long-term survival. The surgical method and lymph node dissection have no significant effect on the survival of patients in whom the histological composition of the micropapillary is ≤5%. This indicates that the micropapillary component has a certain value for the specific surgical approach of early-stage lung adenocarcinoma and prognosis in lymph node metastasis.

In this study, 390 patients with non-small cell lung adenocarcinoma who underwent pulmonary nodule surgery were grouped according to the proportion of micropapillary histological components. Through multivariate and survival analyses, we found that the long-term survival of patients with > 5% micronipple components was significantly lower than that of patients with ≤ 5% micropapillary components, and the histological component of the micropapillary was an independent risk factor. Tamás Zombori et al. (14) found that the prognosis of patients with acinar lung adenocarcinoma is fairly good, while those patients with solid and micropapillary histological components are more prone to recurrence, metastasis, and an overall worse prognosis. In the study of Jun-ichi Nitadori et al. (15), IASLC/ATS/ERS classification was used to determine that the presence of 5% or more micropapillary components was independently associated with the risk of recurrence in patients treated by lung wedge resection. Katsuya Watanabe et al. (16) studied 1289 patients with lung adenocarcinoma who underwent pneumonectomy from 2008 to 2015. It was found that the risk curve of patients with micropapillary components showed a broad peak within one year after operation, while those without micropapillary histological components showed some gentle peaks approximately two years after operation. In stage I patients, the presence of micropapillary components was associated with a poor recurrence-free survival rate and early recurrence but not in advanced patients. This indicates that patients with micropapillary components have a high risk of early postoperative recurrence, and the risk of recurrence exists for a long time. Even after complete resection of stage I lung adenocarcinoma, micropapillary components are still associated with poor prognosis. The results of this study are similar, and the prognosis of patients without micropapillary components is better than that of patients with micropapillary components. Because of these studies, the micropapillary histological components are of great significance in precisely selecting suitable patients for sublobar resection.

For resectable NSCLC, comprehensive treatment is still based on lobectomy and mediastinal lymph node dissection (5, 6). In the Japanese JCOG0802 study, the overall survival of sublobectomy was significantly better than that of lobectomy in these early patients (more people died of other diseases in the lobectomy group). Although the local recurrence rate is increased, greater preservation of lung parenchyma increases the room for subsequent treatment, such as in cases of disease progression and secondary primary cancer (17, 18). In this study, it was found that when the proportion of histological components of micronipples was different, the survival of patients after different operations was different. When the micropapillary histological components were more than 5%, the survival rate of lobectomy was higher, while in the patients with micropapillary histological components lower than 5%, there was no significant difference in survival rate after different surgical methods. Yao et al. (19) found that patients with subcentimetre lung adenocarcinoma with micropapillary components had a poor prognosis, and wedge resection was associated with a higher risk of recurrence than anatomic lung resection (segmentectomy and lobectomy), which was similar to our results.

Lymph node metastasis is the most reliable indicator of staging and prognosis of patients with lung cancer, but excessive lymph node dissection may increase the time of operation and drainage and may damage the nerve, blood vessel and lymphoid structure in the mediastinum, resulting in increased postoperative complications. In our study, when the micropapillary components were more than 5%, the survival rate after systemic lymph node dissection was higher, while in patients with micropapillary components ≤5%, the survival rate showed no significant change after undergoing different lymph node dissections. Sun et al. (20) analysed the clinicopathological data of 1160 patients with ≤2 cm invasive lung adenocarcinoma who underwent surgery from seven medical institutions from January 2012 to December 2015. It was found that limited mediastinal lymph node dissection was an independent prognostic factor for N2 lymph node metastasis in patients with micronipple and solid components >5%. The recurrence-free survival and overall survival time of patients who underwent systemic lymph node dissection were better than those who underwent limited lymph node dissection. In patients whose sum of micronipple and solid components ≤ 5%, the prognosis of localized lymph node dissection was similar to that of systemic lymph node dissection. This is also similar to our research results.

The results of this study may be based on the following reasons. First, micropapillary lung adenocarcinoma has special structural characteristics. Its tumor cells are small and cuboid, grow in papillary clusters without fibrous vascular axes, and can be attached to the alveolar wall or fall off into the alveolar cavity. Because of its unique “inside-out” growth mode, the tumor cell cluster has a strong invasive behaviour, which spreads easily, is more prone to vascular and interstitial invasion, and is prone to early recurrence and metastasis (21, 22). Second, when the patient has micropapillary histological subtype components, the tumor resection margin is insufficient after sublobectomy, and the tumor is more likely to metastasize and recur. Third, when the patient has micropapillary histological subtype components, the number of lymph node dissection stations and the number of lymph nodes were significantly reduced when the patient underwent limited lymph node dissection, and the lymph node postoperative pathology showed false negatives. No accurate lymph node staging was provided for patients with lung cancer, and postoperative adjuvant treatment was not available in time, which shortened the survival time of patients.

Our research also has some limitations. First, this is a retrospective study, which may lead to limited data and some selection bias. To verify our findings, it is necessary to conduct randomized trials in the future. Second, the sample size of patients is limited. Third, the heterogeneity of the tumours will inevitably have some potential impact on the diagnosis of pathological sections. At present, due to the limitation of intraoperative frozen pathological conditions, many medical centres cannot fully judge the micropapillary histological subtype components according to intraoperative pathology. Although preoperative biopsy can well predict histological composition, the availability of samples is limited, and this invasive procedure may lead to many complications. The relationship between imaging data and micropapillary components needs further confirmation to guide clinical decision-making.

## Conclusions

The micropapillary histological component is an independent risk factor after surgery in patients with ≤2 cm lung adenocarcinoma. When the proportion of micropapillary components is different, the prognosis of patients is different when different surgical methods and lymph node dissections are performed. Lobectomy and systematic lymph node dissection are recommended for patients with a micropapillary histological composition >5%; sublobar resection and limited lymph node dissection are recommended for patients with a micropapillary histological composition ≤5%. The feasibility of this strategy needs to be prospectively validated in future work. It is believed that more data are needed to better clarify this issue.

## Data availability statement

The original contributions presented in the study are included in the article/supplementary material. Further inquiries can be directed to the corresponding authors.

## Ethics statement

The studies involving human participants were reviewed and approved by the Affiliated Provincial Hospital of Anhui Medical University. Written informed consent for participation was not required for this study in accordance with the national legislation and the institutional requirements.

## Author contributions

LX: Conceptualization, methodology, software, investigation, writing - original draft, writing – review, and editing. HZ: Conceptualization, methodology, investigation, histological evaluation. GW: Methodology, software, investigation. ZH: Methodology, software, investigation. RX: Visualization, investigation. XS: Visualization, investigation. MW: Visualization, investigation. TL: Writing - original draft, writing – review, and editing. MX: Funding acquisition, writing – review, and editing. All authors contributed to the article and approved the submitted version.

## Funding

This work was supported by the grants from the National Natural Science Foundation of China and Key research and development projects in Anhui Province (NO.81973643and 202004j07020017).

## Acknowledgments

The authors thank Yuehong-Shen for their help with data collection and preparation of figures.

## Conflict of interest

The authors declare that the research was conducted in the absence of any commercial or financial relationships that could be construed as a potential conflict of interest.

## Publisher’s note

All claims expressed in this article are solely those of the authors and do not necessarily represent those of their affiliated organizations, or those of the publisher, the editors and the reviewers. Any product that may be evaluated in this article, or claim that may be made by its manufacturer, is not guaranteed or endorsed by the publisher.
